# Identifying Future Study Designs and Indicators for Somatic Health Associated with Diets of Cohorts Living in Eco-Regions: Findings from the INSUM Expert Workshop

**DOI:** 10.3390/nu16152528

**Published:** 2024-08-02

**Authors:** Dominika Średnicka-Tober, Rita Góralska-Walczak, Klaudia Kopczyńska, Renata Kazimierczak, Michał Oczkowski, Carola Strassner, Friederike Elsner, Lea Ellen Matthiessen, Thea Steenbuch Krabbe Bruun, Beatriz Philippi Rosane, Cesare Zanasi, Marja Van Vliet, Lars Ove Dragsted, Sarah Husain, Camilla Trab Damsgaard, Denis Lairon, Emmanuelle Kesse-Guyot, Julia Baudry, Catherine Leclercq, Lilliana Stefanovic, Ailsa Welch, Susanne Gjedsted Bügel

**Affiliations:** 1 Department of Functional and Organic Food, Institute of Human Nutrition Sciences, Warsaw University of Life Sciences, 02-776 Warsaw, Poland; rita_goralska_walczak@sggw.edu.pl (R.G.-W.); klaudia_kopczynska@sggw.edu.pl (K.K.); renata_kazimierczak@sggw.edu.pl (R.K.); 2 Department of Dietetics, Institute of Human Nutrition Sciences, Warsaw University of Life Sciences, 02-776 Warsaw, Poland; michal_oczkowski@sggw.edu.pl; 3 Department of Food—Nutrition—Facilities, FH Münster University of Applied Sciences, 48149 Münster, Germany; strassner@fh-muenster.de (C.S.); friederike.elsner@fh-muenster.de (F.E.); sarah.husain@fh-muenster.de (S.H.); 4 Department of Nutrition, Exercise and Sports, University of Copenhagen, 1958 Frederiksberg, Denmark; lem@nexs.ku.dk (L.E.M.); bpr@nexs.ku.dk (B.P.R.); ldra@nexs.ku.dk (L.O.D.); ctd@nexs.ku.dk (C.T.D.); shb@nexs.ku.dk (S.G.B.); 5 Department of Agricultural and Food Sciences, University of Bologna, 40127 Bologna, Italy; cesare.zanasi@unibo.it; 6 Stichting Institute for Positive Health, 3521 AL Utrecht, The Netherlands; m.vanvliet@iph.nl; 7 Inserm, INRAE, C2VN, Aix Marseille Université, 13331 Marseille, France; denis.lairon@orange.fr; 8 Inserm, INRAE, Cnam, Nutritional Epidemiology Research Team (EREN), Epidemiology and Statistics Research Center—Paris Cité University (CRESS), Sorbonne Paris Nord University, 93000 Bobigny, France; e.kesse@eren.smbh.univ-paris13.fr (E.K.-G.); j.baudry@eren.smbh.univ-paris13.fr (J.B.); 9 Food and Nutrition Center, Council for Research in Agriculture and the Analysis of the Agriculture Economy (CREA), 00178 Rome, Italy; 10 Section of Organic Food Quality, Faculty of Organic Agriculture Sciences, University of Kassel, 37213 Witzenhausen, Germany; l.stefa@uni-kassel.de; 11 Norwich Medical School, Centre for Population Health Research, Faculty of Health, University of East Anglia, Norwich, Norfolk NR4 7TJ, UK; a.welch@uea.ac.uk

**Keywords:** somatic health, biomarkers, indicators, sustainable and healthy diet, Eco-Regions, Biodistrict, sustainable food systems, INSUM, workshop

## Abstract

Diets, but also overall food environments, comprise a variety of significant factors with direct and indirect impacts on human health. Eco-Regions are geographical areas with a territorial approach to rural development, utilizing organic food and farming practices, and principles and promoting sustainable communities and food systems. However, so far, little attention has been given to quantifying aspects of the health of citizens living in these sustainable transition territories. The project “Indicators for Assessment of Health Effects of Consumption of Sustainable, Organic School Meals in Eco-Regions” (INSUM) aims to identify and discuss research approaches and indicators that could be applied to effectively measure the somatic, mental, and social health dimensions of citizens in Eco-Regions, linked to the intake of organic foods in their diets. In this paper, we focus on the somatic (physical) health dimension. A two-day workshop was held to discuss suitable methodology with an interdisciplinary, international group of experts. The results showed the limitations of commonly used tools for measuring dietary intake (e.g., relying on the memory of participants), and nutritional biomarkers (e.g., variations in correlations with specific intakes) for research understanding dietary intake and the health effects of diets. To investigate the complexity of this issue, the most suitable approach seems to be the combination of traditional markers of physical and mental health alongside emerging indicators such as the microbiome, nutrigenomics, metabolomics, or inflammatory biomarkers. Using new, digital, non-invasive, and wearable technologies to monitor indicators could complement future research. We conclude that future studies should adopt systemic, multidisciplinary approaches by combining not only indicators of somatic and mental health and social wellbeing (MHSW) but also considering the potential benefits of organic diets for health as well as aspects of sustainability connected to food environments.

## 1. Introduction

Food systems are broadly considered a significant entry point for sustainable change [1,2]. While current food production and consumption are strongly contributing to major global environmental, food security-related, and wellbeing challenges [3], sustainable food systems have the potential to mitigate climate change, protect our planet, and sustain public health [4]. In response to this, a novel initiative for sustainable communities, called “Biodistrict” or “Eco-Region”, was created in Cilento, Italy, in 2009, with the objective of promoting the sustainable management of local resources through the implementation of organic farming, agroecology, regenerative agriculture and other sustainable food production, and related sustainable services like eco-tourism [5]. The overall concept of Eco-Regions, based on a territorial approach to rural development utilizing organic food and farming practices and principles and promoting sustainable communities and food systems, is gaining attention globally, and many Eco-Regions are currently emerging in various areas around the world. However, limited focus has so far been given to the citizens’ health aspects of these sustainable transition territories [6].

The research project “Indicators for Assessment of Health Effects of Consumption of Sustainable, Organic School Meals in Eco-Regions” (INSUM) aims to address this important gap by defining suitable study designs and indicators, including somatic and nutritional biomarkers, to be used for future studies on the diet and health nexus in Eco-Regions. The transition from a pathogenic (disease-oriented) to a salutogenic (health-oriented) approach to human health and wellbeing, aligned with the INSUM project’s focus, paves the way for health promotion to become a focus point of development and policies. The promotion of public health as a crucial step to improve individual health and wellbeing could be effectively addressed, i.e., at the school level, by endorsing the importance of school health policies, a healthy school environment, and the provision of safe water, sanitation, and nutrition services [7]. School meal systems are an ideal nexus for all actors and stakeholders in communities to tackle the diet’s role in promoting health. Eco-Region communities are a noteworthy target group to proceed further with this notion and study the effects of the diet and the living environment on health, considering its various dimensions [8].

The INSUM research on health indicators of the transition towards more sustainable and organic diets has been divided into the following health domains: “mental health and social wellbeing” (MHSW) and “somatic health”. Consequently, two workshops with different experts were organized within the project. The first INSUM workshop dedicated to MHSW took place in May 2022 in Münster (Germany), bringing together a multidisciplinary and multinational group of experts, and yielded the first INSUM consensus paper published in January 2023 [9]. The paper presented here summarizes the outcomes of the second INSUM workshop organized in October 2022 in Warsaw and focuses on somatic health indicators. The three major aims of the second INSUM workshop were to (a) exchange experience and evidence on state-of-the-art and emerging methodologies and indicators in the field of dietary intake and somatic health, (b) discuss potential study designs and indicators that would fit well into the setting of an Eco-Region environment, and (c) create a strong, multidisciplinary network of experts with diverse backgrounds in the areas of food science, public health, nutritional physiology and epidemiology, nutritional intake and health, biomarkers of food intake, agriculture, food systems, and environmental indicators of diets, capable of addressing the defined INSUM challenges in future joint collaboration.

## 2. Methodology

Based on the scientific literature on health indicators of diets, the academic network, and the outcomes of the first INSUM workshop on MHSW [9], experts were identified and contacted by the project team. Over 150 invitations were sent out by email. The 2-day hybrid workshop was hosted in Warsaw, Poland, in October 2022. Overall, 27 participants joined the workshop, including representatives of 8 nations (i.e., Denmark, France, Germany, Italy, Poland, Sweden, The Netherlands, and The United Kingdom) with various expertise (i.e., food science, human nutrition, dietetics, nutritional physiology, public health, health science, organic food systems, biomarkers of food and nutrient intake, dietary intervention studies, and nutritional epidemiology).

The workshop agenda comprised a comprehensive “Setting the scene” session, where the INSUM approach to defining health was introduced, followed by the summary of the 1st INSUM Workshop on MHSW, as one of the intertwined health dimensions. That session also provided insights into organic food production in relation to food quality and safety, as well as the links between organic food-based diets and human health. The concept of Eco-Regions was then introduced, highlighting the main gaps in the current Eco-Region-focused research. The “Key areas and positions” session of the workshop started with an introduction to “Positive Health” as a concept for a broad and dynamic perception of health, followed by an overview of existing, new, and emerging (bio)markers used to measure dietary change. Study designs, the biomarkers researched, and the main outcomes of selected (including organic) food-/diet- and health-focused studies—BioNutriNet and OPUS—were also presented. Finally, insights into new digital solutions to monitor indicators of diet, lifestyle, and health were provided. 

Additionally, two Eco-Region-focused projects were briefly introduced to inform the participants of a number of sustainability aspects and approaches to be potentially addressed in future Eco-Region research.

All expert presentation sessions were accompanied by open and guided discussion rounds and wrap-ups. Experts debated current, new, and emerging indicators and tools to measure the effects of dietary transitions towards more sustainable and organic diets, suitable study designs, and their potential and limitations. The major guiding question for the workshop was “How can we test whether Eco-Regions’ communities and food systems which are more local, more sustainable and organic, benefit the health of Eco-Regions’ citizens?”. This article summarizes the experts’ contributions (i.e., Section 4 and Section 5), the main discussion points, and major conclusions and outlook arising from the workshop.

## 3. INSUM Health, Organic Food, and Eco-Regions

### 3.1. INSUM’s Approach to Health

**Figure 1:**
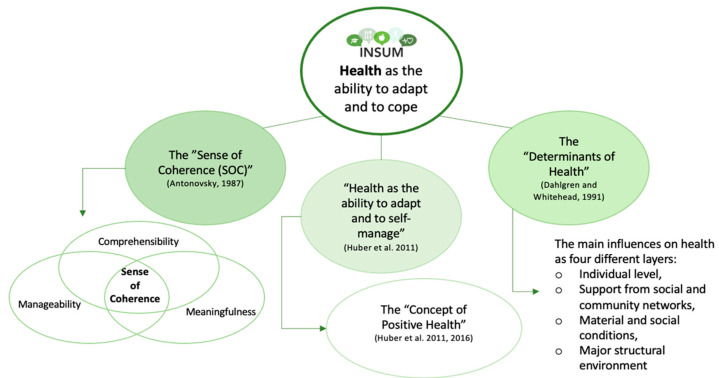
The understanding of health within the project “Indicators for Assessment of Health Effects of Consumption of Sustainable, Organic School Meals in Eco-Regions” (INSUM). Scheme developed based on the 1st INSUM workshop, May 2022. Source: Elsner et al. [9]; Antonovsky, 1987 [18]; Huber et al., 2011 [12]; Huber et al., 2016 [13]; Dahlgren and Whitehead, 1991 [14].

According to the definition of the World Health Organization, health is understood as “a state of complete physical, mental and social wellbeing and not merely the absence of disease or infirmity” [10,11]. This definition takes into consideration three key domains of health (somatic, mental, and social). However, its static character and absoluteness (understood as a complete state of being healthy or not), in our view, do not fully fit today’s society with all its challenges [12]. From a systems perspective, health is not static but rather it characterizes the dynamics of the ability to deal with challenges and be resilient and adaptive [12,13]. Thus, INSUM considers health as the individual’s ability to adapt and cope under the influence of various (internal and external) factors [13,14,15,16,17]. This concept of health was built upon, among others, Aaron Antonovsky’s “Sense of Coherence” [18] (SOC), the “Determinants of Health” [14], and the “Concept of Positive Health” [13,19] (see Figure 1).

### 3.2. Outcomes of 1st Workshop on Social and Mental Health

The first INSUM workshop focused on the current methodologies and indicators in the field of MHSW associated with diets from the perspective of Eco-Regions and proposed potential study designs for the populations of Eco-Regions, such as cohort studies addressing families, including in-depth interventional and/or experimental studies. The main tool to examine MHSW was questionnaires, with their structure and content dependent on the research design, i.e., target groups/participants or distribution channels. The surveys and scales discussed included, among others, the Warwick–Edinburgh Mental Wellbeing Scale [20], the Stirling Children’s Wellbeing [21], the Perceived Stress Scale, and the Spider web tool from Positive Health Concept [22]. The latter is of special interest to the INSUM project since it takes into consideration aspects such as organic and sustainable diets.

Participants of the first INSUM workshop highlighted that mental, social, and physical (somatic) health interactively form the complex of human health and advised they should not be assessed separately. Moreover, the health study designs and indicators should allow the simultaneous measurement of all these health dimensions. Moreover, explorative research designs are required to investigate the complexity of Eco-Regions and diverse study populations have been discussed with the understanding that any age group could be selected in the cohort study [9]. These concepts were elaborated further in the second INSUM workshop.

### 3.3. Somatic Health Perspective

The synonyms of the word “somatic”, according to the literature, include “physical”, “anatomical”, “physiological”, and “clinical-anatomical” integrity. When searching online publication databases for studies on diets or dietary changes vs. somatic health using keywords such as “diet” or ”nutrition”, “somatic health” or “physical health”, and “indicator” or “biomarker”, the majority of the search outcomes include scientific literature focusing on certain diseases or disease risks, i.e., diabetes mellitus, gestational diabetes mellitus, cardiovascular diseases, inflammations, oral medical conditions, obesity, and various other non-communicable diseases. In these studies, “the health status” is assessed based on various results of diagnostic tests from blood, urine, and anthropometry that can be compared to reference value ranges appropriate for population groups in different countries or geographical regions. Presently, there is a wide range of such markers that are generally accepted for diet-related disease diagnosing (i.e., fasting serum glucose level, plasma lipid profile (serum total cholesterol, triglycerides, HDL, and LDL levels), serum electrolyte level, C-reactive protein, plasma hormones, basic urine tests, and BMI) [23,24,25,26].

The INSUM’s approach to health as the individual’s ability to adapt and to cope, in its somatic dimension, seeks new and emerging areas with the potential to yield novel (bio)markers of health impacts of dietary changes. Among them, those gaining interest include microbiome, nutrigenomics, metabolomics, markers of oxidative status, and biological age. At the same time, in the need to test the various somatic health markers in relation to numerous lifestyle factors, novel digital health technologies emerge. We touch on the topic of these various markers and measuring tools and devices in the following sections of the article.

### 3.4. Organic Food

In accordance with Regulation (EU) 2018/848 of the European Parliament and the Council of 30 May 2018 on organic production and the labeling of organic products [27], “Organic production is an overall system of farm management and food production that combines best environmental and climate action practices, a high level of biodiversity, the preservation of natural resources and the application of high animal welfare standards and high production standards in line with the demand of a growing number of consumers for products produced using natural substances and processes”. Organic production systems thus aim to deliver publicly available goods while contributing to the protection of the natural environment, animal welfare, and overall rural development. 

Crops in organic farms are grown without the use of synthetic plant protection agents (insecticides, herbicides, and fungicides), highly soluble synthetic nitrogen, phosphorus and potassium fertilizers, or synthetic growth regulators. Soil fertility in organic farms is maintained using natural organic fertilizers and green manures, along with diversified crop rotation. Plants are protected against pests and weeds with the use of biological and mechanical methods. Organic husbandry prioritizes animal welfare by providing appropriate living conditions, including access to open spaces or pastures, enabling the implementation of natural behaviors, and providing high-quality organic feeds without synthetic additives. Organic food processing should be gentle, preferably using biological and physical methods, and should not be deceptive about the true nature of the food. The global organic food market has been dynamically expanding in the last decades [28]. Following environmental and animal welfare considerations, human health is stated by consumers as one of their major motivations for opting for organic foods [29]. According to the EU’s Action Plan, organic farming is amongst the key strategies to produce high-quality and safe foods with a low environmental impact and other sustainable socio-economic benefits [27].

Within the last 30 years, more than 500 studies analyzing various quality and safety aspects of organic foods have been published [30,31]. Researchers have been looking into differences in composition, i.e., mineral compounds, heavy metals, pesticide residues, macro-nutrients, phenolic compounds, and vitamins, comparing organic vs. non-organic foods. Authors of a large meta-analysis summarizing the results obtained from various food composition studies concluded that organic fruits and vegetables are, on average, up to 70% richer in polyphenolic compounds and organic dairy and meat products show more beneficial fatty acid profiles [31,32,33]. Organic foods were also significantly less frequently contaminated with pesticide residues [31,34].

Lower pesticide exposure of organic food consumers, measured by the concentrations of pesticide metabolites in urine, is now well documented [35,36]. Moreover, several researchers have reported inverse links between organic food consumption and other negative health outcomes (e.g., cancers and metabolic syndrome) [37,38]. Organic diets were also associated with several indicators of a healthy and sustainable diet and lifestyle (i.e., a more plant-based diet and higher physical activity), as well as lower incidences of overweight and obesity [38,39]. One of the most comprehensive studies in this area carried out to date, BioNutriNet, aiming to characterize organic food consumers and investigate the relationships between organic food consumption and overall dietary patterns, food pesticide exposure, health, and impacts on resources and the environment, will be presented in one of the following sections of this paper, focused on study designs and indicators.

Even though the cited studies point to positive health effects related to organic diets [40], further research in different contexts is important to investigate and clarify underlying factors and improve the scientific level of proof. Moreover, the majority of studies looking into the impacts of organic diets focus exclusively on the somatic dimension and on classical disease risk rather than health markers [39,40,41,42].

### 3.5. Eco-Regions—Concept, Aims, Research Gaps

Biodistricts, also termed Eco-Regions, Organic Districts, or Eco-Districts, are territories where farmers, citizens, public authorities, and other local actors realize a formal agreement aimed at the sustainable management of local resources, based on the principles and model of organic farming and the agroecological best practices in order to boost the economic and socio-cultural development of their community [43,44]. Values and practices in Eco-Regions, starting from bottom-up initiatives, revolve around organic farming, agroecology, regenerative agriculture, and other sustainable food production methods and related sustainable services like eco-tourism. They rely on a committed and inclusive community able to effectively manage the interactions between the different aspects contributing to sustainable development [8].

The concept of a Biodistrict is highly coherent with policies, matching most of the objectives, among others, of the EU Green Deal (25% organic land in the EU by 2030) [45]; the EU Organic Action Plan [46], in particular Action 14; the EU long-term Vision for the Rural Areas [47]; the UN Food and Agriculture Organization’s definition of sustainable diets [48]; and UN Sustainable Development Goals. They are in line with the main policies and strategies related to Sustainable Food Systems Development, providing a favorable context for developing, among others, sustainable tourism, sustainable agricultural practices, sustainability in the entire food system, and community participation and strengthening rural–urban links.

Research on Eco-Regions has thus far been primarily focused on the organic approach and promotion of local value chains [6,8,49,50]. Biodistrict researchers point to the main research gaps and needs being in the area of the impact of Biodistricts on economic sustainability, agronomic performance, environmental sustainability, supply and value chain performance, and finally, social sustainability, including health, food security, food safety, gender issues, worker conditions, and resilience. All of these should be supported by reliable research data to enhance their dissemination and sustainable development.

## 4. Positive Health, Indicators of Dietary Change, and Study Designs

### 4.1. Positive Health

Dr. Marja van Vliet opened her talk by showing an earlier research project about the somatic health effects of an organic diet. In that study, chickens fed organic feed were shown to be able to better overcome immunological challenges compared to chickens fed a conventional diet [51]. Based on the current WHO definition of health [10], it was impossible to conclude whether the observed response reflected a healthier condition. Over the years, more concerns were expressed about the static character of the WHO definition of health [52]. Therefore, an expert group led by Dr. Machteld Huber proposed a more dynamic concept of health: ‘health as the ability to adapt and self-manage’ [12]. According to this concept, one’s health is reflected by the level of resilience and the ability to overcome challenges during the life span. 

For public health purposes, the new, dynamic concept of health was operationalized into the concept of Positive Health (PH) [13]. Positive Health encompasses six dimensions: body functions, mental wellbeing, meaningfulness, quality of life, social and societal participation, and daily functioning [13]. PH shows that health is a broad and complex construct in which dimensions can affect each other. It implies that health is not just a state of ease or disease. Despite having (for example) a chronic disease, people can still be relatively healthy in other dimensions. Overall, the concept of PH, strongly shaping the INSUM approach to health, highlights that when studying the somatic health effects of a diet, these effects should always be regarded in a broader perspective.

### 4.2. Biomarkers of Dietary Change

Professor Lars Ove Dragsted spoke about new and emerging biomarkers of dietary intake. He pointed out the problem of the current instruments for determining food intake in nutrition research being inherently subjective, based on volunteers’ memory, willingness to answer, knowledge about foods, etc., which causes potential bias in the dietary intake data. This problem concerns not only the reporting of the amount of food items that have been consumed and the ingredients used for the prepared dishes but also the portion size and underlying databases. There are attempts to solve these problems through the use of relevant questionnaires (FFQs, 24-h and multiple-day dietary recalls), adjusted to the purpose of the study and the specificity of the surveyed population. However, imprecise recording of food consumption data contributes to the under- or overestimation of food intake [53].

Objective biomarkers of food intake may provide a more accurate assessment [54]. Biomarkers are based on the chemical analysis of biological samples (i.e., saliva, urine, feces, blood, and hair) from study volunteers. They can be used for the purpose of characterizing exposure (what you eat), susceptibility/resilience (health), or effect (change), and explaining how these interact. The workshop experts discussed the usefulness of omics techniques, such as metabolomic analyses, to identify and quantify biomarkers of diet, dietary changes, and health. These biomarkers have several limitations. Firstly, currently, there are only a few validated biomarkers that may be used for the quantitative estimation of food intake. The roadmap for finding and validating biomarkers has been defined in previous projects such as FoodBAll, and candidate biomarkers covering many foods and food groups are already known. However, for complex foods or whole diets, multiple biomarkers must be combined to provide an overall qualitative estimate of whether a subject has consumed them [55]. This estimate of compliance can in turn be used statistically to evaluate the biological effects of a food or diet and measures of sustainable diets would therefore, in principle, be possible. In summary, biomarkers cannot substitute subjective dietary instruments at this time; however, biomarkers can be used to classify subjects by their intake of specific foods or diets.

### 4.3. Oxidative Stress Biomarkers

Sara Hussain introduced the topic of oxidative stress and its biomarkers. The term “oxidative stress” was first coined by Dr. Helmut Sies as an imbalance between the production of oxidants, reactive oxygen species (ROS), and antioxidant defenses that may result in damage to the biological system. After decades of research in this field, oxidative stress is postulated to be understood as “a state in which the pro-oxidative processes overwhelm cellular antioxidant defences due to the disruption of redox signalling and adaptation” [56]. Oxidative stress plays a vital role in the pathogenesis of many lifestyle-related diseases (i.e., diabetes, obesity, cardiovascular diseases, neurodegenerative diseases, and cancers) [57]. Sara Hussain pointed out the importance of lifestyle (including diet) factors in strengthening the body’s antioxidant defense system and preventing lifestyle-related diseases [58,59]. Oxidative stress biomarkers can be used to assess the severity of such diseases. Identifying oxidative stress biomarkers responsible for disease development could help in understanding its progression. However, due to their unstable nature, the direct measurement of ROS is not a preferred strategy, hence measuring the ROS indirectly or detecting antioxidant levels due to their stable nature are considered a more reliable approach [60]. Numerous techniques, ranging from DNA oxidation to protein damage, lipid peroxidation, and oxidation of free amino acids, have been developed and used to assess the degree and type of oxidative stress in almost all diseases. One more point that needs to be considered is analyzing the oxidative stress status by evaluating multiple biomarkers for a more comprehensive understanding of the diseases and to find their root cause. 

One of the existing multiple-biomarker-based indicators of oxidative status is the “reserve capacity” of the organism, calculated by integrating several parameters: the marker of oxidative stress (MDA), the antioxidant defense index (i.e., SOD—superoxide dismutase), and non-enzymatic low molecular antioxidants (i.e., GSH). Blood and urine are biological fluids suitable for analyzing oxidative stress indicators. Cells isolated from the blood can be used to study lipid peroxidation (MDA) in their cell membranes. Urine, on the other hand, is relatively easy to obtain non-invasively and can be stored stably for long periods of time. Moreover, it can be used for both oxidative stress indicators and metabolomic analyses [61].

### 4.4. Study Designs and Indicators: OPUS School Meal Study

In her talk, Professor Camilla Trab Damsgaard gave an overview of the design, markers investigated, and main results of the OPUS School Meal Study. The aim of the OPUS study was to investigate the impact of climate-friendly Nordic school meals on dietary intake and nutritional status, growth, body composition, bone health, cardiometabolic markers (1º outcome: MetS score), school performance, attention and wellbeing, sleep and physical activity, and social and cultural changes. The study was a cluster-randomized crossover trial among 8–11-year-old pupils at nine Danish schools in 2011–2012 [62]. All pupils of the third and fourth classes were invited, 82% participated (*n* = 834), 14% had overweight or obesity, and 8% dropped out. In this study, children received freshly made New Nordic Diet-school meals or the usual packed lunch from home (control), each for three months. The meals were rich in fruits, vegetables, and fish. The study started with a two-hour baseline interview (socioeconomic status, habits, etc.), including instructions. Outcomes included seven-day dietary records, seven-day physical activity and sleep by ActiGraph, anthropometry, dual-energy X-ray absorptiometry (DXA) scans, blood pressure, a number of blood biomarkers (cardiometabolic markers such as total cholesterol, LDL, HDL, and triacylglycerol; glucose and insulin; inflammatory markers such as CRP, IL-6, TNF-alpha, and adiponectin; appetite hormones such as ghrelin and leptin; markers of growth and bone conditions such as IGF-1, IGF-BP3, osteocalcin, and PTH; and diet and nutrition status such as 25-hydroxy-vitamin D (vitamin D status), whole blood fatty acid composition (*n*-3 long-chain polyunsaturated fatty acids (LCPUFA) status and fish intake), hemoglobin (iron status), ferritin (iron status), and alkylresorcinols (wholegrain intake)), and tests of cognition/school performance [62]. Compared to the control, the provided school meals increased children’s fish intake and whole-blood *n*-3 LC-PUFA and intake of vegetables, protein, and dietary fiber and reduced fat intake [63]. The school meals reduced diastolic blood pressure, total and HDL cholesterol, triacylglycerol, and insulin compared to the control, with no difference in BMI, z-score, or fat mass index despite a small increase in waist circumference [64]. Children receiving New Nordic Diet-school meals also had improved reading speed and correctness, but more attention test errors [65]. Future studies could further investigate gut microbiota, heart rate variability, stress, body image, more specific cognitive tests, quality of life, and effect modifiers such as sex, genotype, ethnicity, socioeconomic status, and parental education.

### 4.5. Study Designs and Indicators: BioNutriNet Study

Professor Denis Lairon presented the design, markers researched, and major outcomes of the BioNutriNet study on organic food and health. He pointed out that although the number of organic food consumers is markedly rising, only small-scale studies have so far described the profiles of “organic consumers”, actual food and nutrient intakes, or diet-related health indicators. The NutriNet-Santé Cohort study aimed to fill this gap by introducing the BioNutriNet project. It aimed to characterize organic food consumers and investigate the relationships between organic food consumption and overall dietary patterns, food pesticide exposure, health outcomes, purchase costs, and impacts on natural resources (land, energy use) and climate (greenhouse gas emissions—GHGs). State-of-the-art statistical data treatments or modeling were performed. The project was funded by a public grant from the French Ministry of Research for 2014–2018 and yielded 22 scientific publications published in 2014–2021.

This French study (www.etude-nutrinet-sante.fr, accessed on 28 June 2024) on organic food consumption and health, managed by EREN team-INRAE (coordinated by E. Kesse-Guyot), aimed to investigate the relationships between nutrition, lifestyle, and health outcomes in a large adult cohort. It is a web-based prospective cohort study that was launched in 2009 with adult volunteers (170,000 subjects in the cohort in 2021) and about 10 years of follow-up (ongoing). Individual data from questionnaires were collected annually through a dedicated secure HTML interface and accompanied by the collection of some biochemical samples (fasting blood and morning urine). Clinical examinations have been performed in a subsample. The registration of health outcomes and validation have been performed (yearly and occasionally).

The following methods were used in the BioNutriNet project: daily dietary intakes (3 × 24 h dietary records using photographs for portion sizes including seven options, self-registered and checked by dieticians) were used for dietary intake assessment. General diet questionnaires or organic FFQ questionnaires were based on 264 food items, with five organic food consumption levels categorized: never, sometimes, half-of-the-time, frequently, and always (scored as 0, 0.25, 0.50, 0.75, and 1, respectively). Diet quality score calculations were based on PNNS-GS scores for adherence to national food-based dietary recommendations and PANDiet scores with respect to nutrient and fiber intake requirements. Daily nutrient intakes were estimated using a national food composition database (3000 food items) [38].

Dietary pesticide exposure via plant food intake (by far the most contaminated food groups) was determined for pesticide residues estimation, using 25 selected pesticides (considering both their frequency of detection above the MRLs (Maximum Residue Levels) and their ADI (acceptable daily intake)). Food contamination data were derived from the CVUA Stuttgart (Chemisches und Veterinäruntersuchungsamt) database accounting for farming practices. For the 180 plant ingredients that were both important constituents of the 264 food items and available in the CVUA database, contamination values in organic and conventional modes were attributed. For each pesticide, the estimated daily intake (EDI) (in µg/kg bw/d) under lower- and upper-bound scenarios was calculated using recommended methods [38,66].

Urine samples were analyzed for pesticide residue detection in a group (nested study) of “organic consumers” (mean proportion of organic food (in g/d) in the diet = 67%) and “non-organic consumers” (mean proportion of organic food (in g/d) in the diet = 3%), matched on all characteristics and consumption of food groups. Analyses of pesticide residues (metabolites in urine, markers of organophosphates, and synthetic pyrethroids) were measured as markers of pesticide exposure in low and high “organic consumers” [38]. Metabolomic analysis (NMR/MS of metabolome profiles) was also performed.

Blood samples were analyzed for metabolic syndrome—a major CVD risk factor associated with central obesity, hypertension, and the dysregulation of glucose and lipid metabolism. The fasting blood glucose, triglycerides, cholesterol (T, HDL, and LDL), S-D tensions, and waist circumference were also assayed [38].

For nutritional status, a subsample of “organic consumers” and “non-organic consumers” was selected (nested study), matched on all characteristics and consumption of food groups. Levels of magnesium, iron, copper, cadmium, fat-soluble micronutrients (α and β-carotene, lycopene, lutein, zeaxanthin, and β–cryptoxanthin), vitamins A and E, and fatty acids (all from C8 to C22) in plasma were assayed [38].

Risk assessment was performed for overweight and obesity, metabolic syndrome, type 2 diabetes, and cancers such as breast, prostate, skin, colon–rectum, non-Hodgkin, and total lymphomas [38].

For food pesticide exposure and chronic diseases, the following focus groups were included in the follow-up: postmenopausal women, for 4.8 y: incidence of new breast cancers; adults, for 5.95 y: incidence of new type 2 diabetes. The estimation of food exposure to 25 active substances was determined (see above) as mixtures (extracted using non-negative matrix factorization) and adjustments for confounders [67,68].

The main results from this study indicated that regular consumers of organic products exhibit specific socio-demographic characteristics (higher education levels, more physical activity, less smoking, and higher income), with a healthier dietary pattern (more plant-based foods), better-fitting food-based and nutritional recommendations, and lower frequency of overweight and obesity, and have a significantly reduced probability of cardiovascular disease risk and lower risk of type 2 diabetes and cancers. They consume much fewer synthetic pesticide-contaminated foods and have significantly less pesticide residue in their urine. Moreover, their dietary patterns have less of an impact on natural resources (land and energy) and GHGs. Thus, they show better compliance with the sustainable diet concept (cf. FAO definition, 2010) and the UN One Health concept [38].

Even though organic food consumption in high-income countries is often attributed to personal choice/behavior, it should be recognized that severe inequities in societies, associated with more limited access of economically challenged individuals to health-promoting foods and other resources, may contribute to poorer health and sustainability outcomes [69].

### 4.6. Future Monitoring of Indicators

The aim of this talk by Dr. Michał Oczkowski was to present an overview of new digital solutions that can be applied to monitor indicators of diet, lifestyle, and health. Digital solutions offer great opportunities to collect information, allowing for health status monitoring and encouraging individuals to improve their dietary behaviors [70]. Nowadays, smartwatches and smartphones with dedicated applications offer easy-to-use solutions to measure indicators, e.g., vital signs, skin temperature, and sleep and activity patterns, and such solutions are willingly used by consumers in everyday life. Aside from traditional monitoring systems, advanced high-tech wearable devices are being developed [71]. Novel types of continuous, non-invasive, and wearable molecular sensing devices are emerging, bringing new options for next-generation personal portable health monitoring [72,73]. They are based on, i.e., skin- and biofluid measurements, and show an increasing potential for the non-invasive quantification of biomolecules in sweat, saliva, and other body fluids. Examples include a wearable nutritional tracker system equipped with a sweat sensor that can facilitate nutritional screening such as vitamin C levels in sweat [74,75,76] or wireless, intraoral hybrid electronics for the real-time quantification of sodium intake for hypertension management. Researchers recognize the increasing role and potential of smart wearable devices in the remote screening and diagnosis of common cardiovascular diseases (e.g., arrhythmias) and in the management of patients with established cardiovascular conditions (e.g., heart failure) [77]. Such innovative wearable devices and mobile electrochemical sensors are undoubtedly promising new candidates to bridge the gap between digital and biochemical analyses for health status indicators. However, limitations such as device accuracy and clinical validity, a lack of standardized regulatory policies, aspects of patient data/privacy, and considerably high costs still hinder the widespread adoption of many of these technologies in clinical practice [77].

Many of the available digital solutions are also designed to provide feedback to motivate and engage people, e.g., improving their physical activity, limiting sedentary behavior [78], improving dietary behaviors, or inducing other lifestyle-related changes, with the potential to result in significant health outcomes [79,80].

## 5. Eco-Regions and Sustainability

### 5.1. Study in Cilento Biodistrict

Dr. Lilliana Stefanovic introduced the study undertaken in the Cilento Biodistrict in the Campania region of Italy, employing the case study design backed by an actor-oriented approach, to disclose the perceived contribution of the Biodistrict to the UN Sustainable Development Goals (SDGs) [81]. Fourteen key actors were interviewed in this study to uncover the perceived outcomes at individual, communal, and ecological levels, and one focus group was performed. While the ecological outcomes revealed corresponded to those reported in the literature, the individual and communal levels uncovered a wide range of multifaceted outcomes spanning quality aspects of organic products, direct producer–consumer links, collaboration and networking over job creation accompanied by the reduction in rural exodus, the valorization of activity and higher wellbeing, and quality of life. The latter outcome was linked to living a full life, working with other people in a natural environment, and having a quieter and slower-paced lifestyle centered around the Mediterranean diet. The contribution to 16 SDGs was perceived to varying extents, with SDGs 12 and 15 perceived to be addressed most distinctively by the Biodistrict, followed by SDG 5, SDG 11, SDG 4, and SDG 2. Follow-up studies are needed to gain deeper insights into the perception of wellbeing and quality of life in Biodistricts and their SDG contribution.

### 5.2. MET Biodistrict—START UP BIO MET Project

Dr. Catherine Leclerq briefly introduced the START UP BIO MET project launched in 2022 in one of the Italian Biodistricts—Maremma Etrusca e Monti della Tolfa (MET) [82]. MET is located in the Latium region and encompasses over 50 farms spread across four municipalities. The activities of the START UP BIO MET project derive from game-based experiential workshops conducted by The Council for Agricultural Research and Economics (CREA). Within the project, local stakeholders, including organic farmers, identified the lack of economic sustainability as the main challenge of MET. It was suggested to be caused by the administrative burden related to organic certification, the higher cost of organic food, and competition with local supermarkets (cheaper and more convenient). Solutions to this problem proposed by stakeholders included, among others, the promotion of local consumption of Biodistrict products through sensory and nutrition education and local procurement through school catering. The proposed activities included a nutrition survey in primary schools (body weight, lifestyle, food habits, and Mediterranean diet), an assessment of attitudes to organic food, training for primary school teachers to perform nutritional education in their classes considering local food products, pre- and post-questionnaires to measure attitude changes, sensory education (theoretical and practical lessons, sensory testing during public events), an assessment of school menus’ sustainability by calculating the carbon/water/land footprint, modeling of more sustainable menus using local ingredients, and communication (video storytelling). The START UP BIO MET project is testing a multidisciplinary approach where food and nutrition research can contribute to increasing the sustainability of a local food system.

## 6. Discussion

Eco-Regions are gaining growing attention and interest, being increasingly recognized as the areas where sustainable development strategies are implemented [83]. At the same time, the health aspects of Eco-Region populations were identified as one of the important gaps in the Eco-Region research carried out to date. Very interesting results on the potential of Eco-Regions to contribute to SDGs, empower farmers, etc., but also pointing to the improved wellbeing and life quality of Eco-Region citizens, presented by Dr. Stefanovic, show how promising these regions are in terms of providing a unique environment with the potential to impact the health and wellbeing of their population [81]. Here, the Positive Health concept, encompassing six interrelated dimensions of health (body function, mental wellbeing, meaningfulness, quality of life, social and societal participation, and daily functioning) appears to be especially relevant to approaching and assessing health impacts of the overall sustainable living environment in these regions [13]. 

It was pointed out that the majority of the Eco-Region-focused research carried out to date has addressed the “state of the art” and “performance” achievements rather than the changes, development process, and dynamics [6,8,49,50]. To look into the long-term impact of diet and environment and their changes, a longitudinal approach was suggested for future research. The outcomes of such longitudinal studies were perceived as important to serve policymakers to assess the potential and directions of developing Eco-Region territories.

Motivated by the Positive Health concept [13,19], Aaron Antonovsky’s “Sense of Coherence” [18] (SOC), and the “Determinants of Health” [14] introduced during the workshop, the participants turned the view on health from the well-established pathogenic (disease-oriented) perspective and the strong separation of physical health from other health dimensions (i.e., MHSW) to a salutogenic (health-oriented) and multidimensional approach. INSUM’s approach to health was introduced as a dynamic concept of being able to deal with challenges and being resilient and adaptive. This approach motivated a discussion on whether measuring health requires a challenge in place, i.e., to see how the organism copes and adapts, which is a standard approach in many studies (i.e., on immune system responsiveness) [51]. The difficulty of measuring the “readiness to act” in a healthy human, not being posed to a specific challenge, was pointed out. Another important point of discussion touched upon the need to select health indicators and biomarkers sensitive enough to measure the health impacts of often subtle dietary changes, especially in healthy subjects, also considering the very good adaptation mechanisms of the human body (e.g., increasing absorption in the gut/lowering excretion of some compounds when their levels in a diet are low) [84,85].

Organic food was a focus of discussion as an important factor in the Eco-Region and health setting. The workshop participants pointed to significant limitations of the well-established approach of linking organic food composition to health, with a focus on, e.g., concentrations of individual chemical compounds (i.e., phenolics, vitamins, macronutrients, etc.) in certain food items or commodities [31]. Many research studies carried out to date make such a link, neglecting several important health-impact-modifying factors, i.e., food and diet composition, processing, the bioavailability of compounds, their metabolism, and other conditions [86]. The experts agreed that the number of organic diet vs. health studies carried out to date is limited, and the existing ones were mainly focused on the assessment of pesticide exposure (metabolites in urine) or a limited number of disease risk factors rather than health (bio)markers, and hardly any touched upon somatic and MHSW dimensions and/or their interrelations [39,41]. Thus, the employed research approaches have, so far, been answering complex health questions only to a very limited extent, proving a great need to approach this complex topic responsively in future research. The discussed limitations, gaps, and lessons learned provide a good basis for future studies.

Professor Lairon presented the design, markers researched, and major outcomes of one of the most sizeable and complex studies (BioNutriNet), investigating the relationships between organic food consumption and overall dietary patterns, food pesticide exposure, selected health/disease outcomes, and impacts on resources and the environment [38]. Both Professor Lairon and Professor Damsgaard provided insights and opened a discussion on a broad battery of biomarkers and indicators employed in organic food- and dietary change-focused studies. This also sparked a discussion on the necessity to adjust biomarkers to relevant target groups (adults vs. children and adolescents [87], with some markers having different roles in adults and children, e.g., insulin in children as a growth marker). Potential areas of interest for future studies in children, based on OPUS research presented by Professor Damsgaard [62,63], were also indicated, including gut microbiota, heart rate variability, stress, body image, and more specific cognitive tests. The necessity to take into account and properly adjust for sociodemographic variables (sex, genotype, and parental education) and lifestyle effect modifiers (physical activity, smoking, alcohol, etc.) to identify the specific impact of organic diet was also pointed out by the experts and discussed.

To measure the effects of diet or dietary changes on health, it is crucial to be able to precisely assess dietary intakes and their change in dietary patterns. This area of research was recognized as dynamically evolving, searching for precise solutions to replace subjective and often biased dietary records with novel, effective, and objective tools and indicators [55]. The discussion, motivated by the presentation of Professor Dragsted, covered the aspect of whether novel metabolomic biomarkers of dietary intake can replace standard questionnaires at this stage. The workshop participants agreed that the substitution of traditional dietary records with biomarkers of food intake is not yet possible. However, it is possible to classify subjects by their intake of specific foods, intake of a group of signature foods for a diet, and microbial metabolic response. This research area was confirmed to be under rapid development, showing significant improvement in the coverage of single foods and food groups with (new and) validated biomarkers of food intake and improved development of methodology for combining biomarkers, thus presenting significant potential for a massive step in the precision of the outcomes of future diet and health research studies [55,88]. Currently, traditional questionnaires can be combined with and supported by emerging biomarkers rather than be substituted by them. Professor Dragsted underlined the exploratory character of metabolomics, at this stage providing an increasing number of qualitative data (i.e., presence of certain foods in the diet), but not yet quantitative (quantity of a certain food item eaten). Much is also still to be done regarding the possibility of considering the individuality of metabolism and metabolites in diet vs. metabolome research.

Focusing on Eco-Regions and organic diets, it was also discussed whether metabolomic approaches could be employed to examine citizens’ exposure to various environmental and other contaminants. A tremendous and dynamic development of this branch of research has been recognized, with the potential for identifying and measuring a broad range of indicators.

The experts also discussed microbiome research as a dynamically developing field and a promising source of indicators and biomarkers of dietary changes. Rapid changes in the host’s gut microbiome and metabolomics profile related to exposure to different dietary patterns were previously demonstrated [89]. On the other hand, during the discussions, it was also pointed out that the microbiome characteristics, as well as its dynamics and dependence on a variety of diet and lifestyle factors, are not easy to assess and interpret in relation to health. It was recently shown that the gut environment (i.e., segmental transit time and pH) explained more variations in gut microbiome and urine metabolome than dietary macronutrients or personal characteristics [90]. This suggests that the gut environment is key for understanding the individuality of the human gut microbiome composition and metabolism [90]. 

The influence of the gut microbiome on metabolomic assessments, as an important confounder and limitation that needs to be considered, was also discussed. Differences in an individual’s microbiome depending on their health/certain diseases [91] were reported to have an impact on the microbiome reaction (reflected in metabolomic assessment) to what is being eaten. Work on certain disease models was acknowledged as necessary to correct for this important confounder.

Despite these concerns, the experts indicated that gut microbiome characterization is an interesting aspect to be included in future Eco-Region, diet, and health studies. Moreover, considering the increasing evidence of the bidirectional communication between the central nervous system and gut microbiota (the gut–brain axis) [92], such a research focus would allow for combining somatic and mental dimensions of health [93,94,95].

Sarah Hussain’s presentation initiated a discussion on the biomarkers of oxidative stress as candidates for somatic health indicators of dietary changes. Biomarkers of oxidative stress were recognized by the experts as important tools in the assessment of disease status and the health-enhancing effects of antioxidants in humans [61]. The impact of lifestyle and diet on oxidative stress levels was underlined [59]. The experts agreed that studies in this field may be important to gain a better understanding and manage/prevent certain diseases. The need for selecting an appropriate combination of oxidative stress biomarkers was pointed out. The limitation of a lack of reference values for many existing oxidative stress/antioxidant status indicators was mentioned, pointing to the need to measure changes/dynamics and differences rather than absolute values.

Three strategies to measure oxidative stress were introduced, including direct and indirect measurements of the RO and the measurement of levels of antioxidant enzymes and other redox molecules, which serve to counterbalance ROS generated in the cell. The limitation of ROS being very unstable and difficult to measure was underlined [96]. Thus, the combination of indirect ROS measurement, via the assessment of ROS-generated damage to biomolecules (protein, DNA, RNA, lipids, and others) and antioxidant reserve, was discussed as a relevant approach [61]. The need for evaluating oxidative stress status by looking at multiple biomarkers (oxidative stress profile) for a more comprehensive understanding was agreed on. The potential of using metabolomics to study the effect of foods and diets on biomarkers, e.g., exposure to pesticides and biomarkers of oxidative damage, was also discussed [97].

The experts agreed on the need to use a battery of (many) sensitive indicators and biomarkers to measure the somatic health impact of dietary changes. Looking at their patterns and combinations is important. At the same time, workshop participants pointed out that searching for novel markers (i.e., in the field of metabolomics, microbiome, and nutrigenomics) and their interrelations can compete with the strategy of using a combination of well-established, existing, traditional markers, and based on them, making predictions with the help of sophisticated mathematical models. However, it was pointed out that the same markers can be used in different combinations to answer different questions, which should be carefully considered. Also, mathematical models need to be carefully validated in independent studies. Moreover, a model should be limited to absolutely necessary factors only. Setting a very specific question and running a model with only necessary factors was recognized as a possibility to bring reliable outcomes, but at the same time, was considered a challenge in rich and multifaceted Eco-Region settings.

The aspect of limitations related to study blinding was also briefly discussed by the workshop participants [98]. Blinding in dietary intervention studies is often considered a challenge since the foods within the intervention and control groups might look and taste different. The aspect of consumers’ awareness of what they eat and how they feel about it (e.g., trust in health-promoting values, the quality and safety of organic foods, and feeling safe and good about eating organic) were discussed as factors of potentially significant influence on the answers to many of the Positive Health questions. This would not be an issue with more objective (bio)markers such as pesticide metabolites in urine.

The potential for employing novel digital solutions to monitor indicators of diet, lifestyle, and health—from mobile apps in smartwatches and smartphones to advanced high-tech wearable devices—was also discussed [77]. Their usefulness to collect dynamic data, and thus complement classical static indicators, was underlined [99,100,101]. The experts recognized them as potentially powerful tools with advantages not only for health monitoring but also for raising awareness and stimulating/motivating (dietary) behavior changes [102]. Examples of novel types of continuous, non-invasive, and wearable molecular sensing technologies were discussed as promising new candidates to bridge the gap between digital and biochemical analyses for health status and dynamics [73]. However, the need for appropriate selection and adjustment for specific population groups (e.g., children vs. adolescents vs. adults vs. elderly) was also underlined.

Based on the “Eco-Regions and Sustainability” workshop session, a complex study design that focuses on collecting various epidemiological data while assessing the sustainability of Eco-Region communities and the local food systems in Eco-Regions (including aspects of organic food’s sustainable economic benefits and the role of local organic food system in supporting fair distribution of food) and monitoring consumers’ attitudes toward organic food consumption was discussed as a relevant approach to be undertaken in future studies. Moreover, motivated by the approach employed in the BioNutriNet study, the importance of calculating the environmental indicators of Eco-Region citizens’ diets was underlined. According to BioNutriNet study results, not only the characteristics of the organic production system but also (and mostly) the overall dietary patterns (i.e., a more plant-based diet, fewer processed meats) of organic consumers in France were responsible for lower values of the studied environmental indicators (i.e., GHG emissions, energy use, and land occupation) of organic diets [66]. It would be of great interest to investigate this aspect in the Eco-Regions setting, particularly looking into biodiversity indicators for which organic systems may have significant beneficial impacts. 

A further discussion about the possibility and relevance of expanding the Eco-Region concept and its investigation to a metropolitan area was suggested by experts as a point of interest.

**Figure 2:**
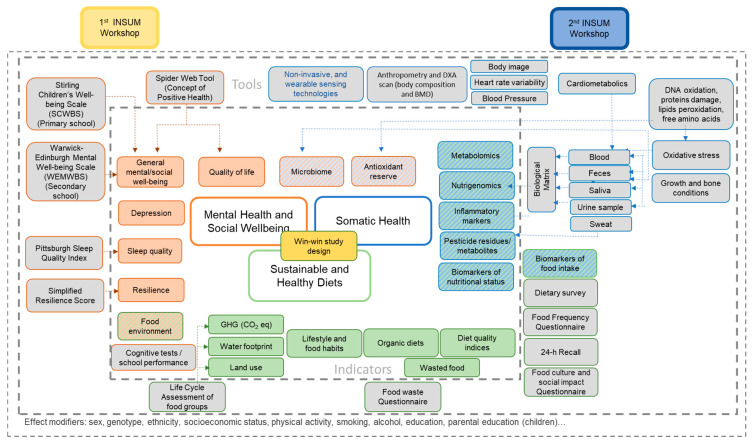
Results of the discussed indicators and tools to assess health. The inner square boundary shows indicators for the three dimensions: Mental Health and Social Wellbeing (orange), Sustainable and Healthy Diets (green), and Somatic Health (blue). The mixed-color boxes symbolize indicators that can be related to more than one category. The outer part displays tools for the assessment of those indicators. Not all indicators were discussed in relation to specific tools. This figure raises no claim for completeness. It serves rather as an overview of the discussed indicators and tools and reflects the main results of the first and second INSUM workshops [9].

Even though during this workshop a strong focus was given to somatic health aspects, it was still highlighted that MHSW and somatic health are closely associated with the complex human health system and highly complementary, and thus should be assessed together in research on the health effects of sustainable and organic diets. As an outcome of the experts’ talks and discussions, Figure 2 presents an overview of tools and indicators for future research on the effects of dietary changes on health, including the interconnectedness of the different health domains. The inner square boundary shows indicators for the three dimensions: Mental Health and Social Wellbeing (orange), Sustainable and Healthy Diets (green), and Somatic Health (blue). The mixed-color boxes symbolize indicators that can be related to more than one category. The outer part displays tools for the assessment of those indicators. This figure, while not necessarily complete, serves as an overview of discussed indicators and tools, reflecting the main results of the two INSUM workshops.

## 7. Workshop Strengths and Limitations

The INSUM workshop on somatic health indicators of dietary changes had its strengths and limitations. The proposed open and explorative approach was a strength, considering that the inclusive, multidimensional, and salutogenic view on human health is still underdeveloped and there is not much known yet about it in association with sustainable and organic diets. Moreover, the group of workshop participants consisted of diverse experts, representing various geographic areas, expertise, and scientific disciplines. This interdisciplinary and international setting clearly contributed to developing a more comprehensive, broad view of the topic of interest, taking into consideration various perspectives. In addition, the hybrid format enabled experts from different countries to participate. At the same time, on one hand, the limited size of the expert group could be seen as a disadvantage that potentially did not allow the gathering of all important views on the topic, but on the other hand, it did support a direct and open discussion where everyone was heard. Furthermore, the overrepresentation of high-income countries by the workshop participants could be recognized as a limitation, not allowing for an appropriate elaboration on the global perspective, i.e., taking into consideration that many low-income countries are challenged nowadays by environmental stresses, including limited access to healthy foods or/and drinkable water. Efforts are needed so that future initiatives in this area include participants from low- and middle-income countries.

## 8. Conclusions and Outlook

The workshop showed that the research on health outcomes of regions affiliated with sustainable development (i.e., Eco-Regions), including sustainable, healthy, and organic diets, is still in a very early stage. At the same time, a comprehensive “battery of tools” (i.e., a combination of different tools and markers from distinct domains) is needed to assess the complex phenomenon of human health in relation to diet and this unique living environment.

Novel and dynamically developing research fields, such as metabolomics, are promising advances to dietary intake assessments and identifying relevant somatic health markers and can be advantageous in offering a better understanding of the complex connection between food/diet and health. However, for many of the novel biomarkers (i.e., from metabolomics, microbiome, and oxidative status/stress fields) further research progress, e.g., validation, and better reference materials are still needed to allow for their successful employment in the context of measuring health impacts of dietary changes. Thus, despite the well-recognized limitations of commonly used traditional dietary intake tools (e.g., the FFQ questionnaire relying on the memory of participants) to investigate the complexity of the issue, it seems it would be most effective to combine traditional markers with novel and emerging indicators. 

New, digital, non-invasive, and wearable technologies used to monitor such health indicators could complement future research. Moreover, future studies should undertake a systemic, multidisciplinary approach, not only combining indicators of somatic health and MHSW but also taking into consideration the potential win–win output of organic diets for health and various sustainability aspects of living in Eco-Regions.

A few aspects were striking and should be considered in future discussions. First, the assessment of changes in healthy participants who are moving towards a more sustainable, organic diet is necessary. Second, we suggest the identification of (bio)markers and indicators that would allow the assessment of even very small differences resulting from such shifts in health. Then, there is a need for adjustment to various confounders, including lifestyle factors, in Eco-Region settings, when the main research focus is on dietary impacts. Fourth, strategies are necessary to overcome/minimize the limitations of both traditional and novel dietary intake assessment tools. Finally, researchers must determine how the somatic and MHSW dimensions should be integrated, and which tools to use to allow for an exploration of health as a whole.

Altogether, taking into consideration all limitations and strengths of the workshop, its outcomes set a good baseline for international, interdisciplinary collaboration and for the development of future research to serve the needs and demands of regions affiliated with sustainable development.
